# Supplementary material for: A Worldwide Analysis of Adipose-Derived Stem Cells and Stromal Vascular Fraction in Orthopedics: Current Evidence and Applications
Source: J Clin Med. 2023 Jul 17;12(14):4719. doi: 10.3390/jcm12144719 (PMC10380598; doi:10.3390/jcm12144719)
Supplement: Supplementary file 1 [file jcm-12-04719-s001.zip › jcm-2403845-supplementary.pdf]

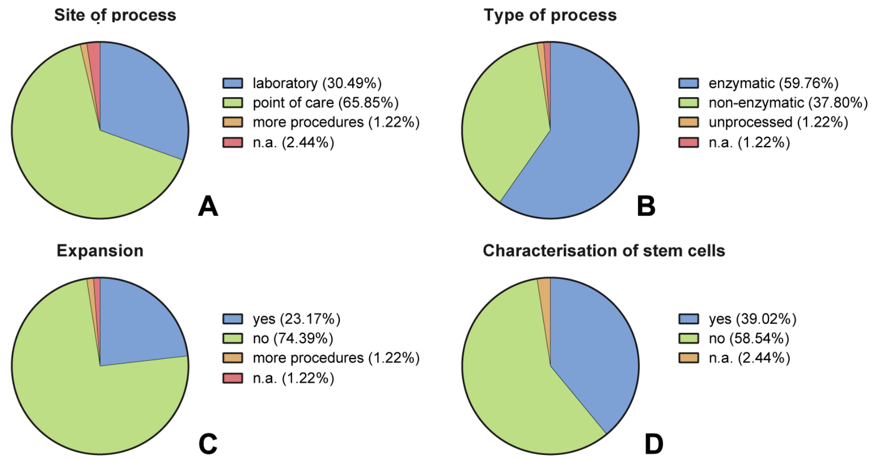

Figure S1. Relevant information on site (A), type of processing (B), expansion (C), and characterization (D) of the mesenchymal stem/stromal cells used in the included studies.
